# Up-Regulation of Long Non-Coding RNA AB073614 Predicts a Poor Prognosis in Patients with Glioma

**DOI:** 10.3390/ijerph13040433

**Published:** 2016-04-19

**Authors:** Lei Hu, Qiao-Li Lv, Shu-Hui Chen, Bao Sun, Qiang Qu, Lin Cheng, Ying Guo, Hong-Hao Zhou, Lan Fan

**Affiliations:** 1 Department of Clinical Pharmacology, Xiangya Hospital, Central South University, Changsha 410008, China; hu773589905@163.com (L.H.); lvqiaoli2008@126.com (Q.-L.L.); scy_csu2016@163.com (B.S.); guoying881212@csu.edu.cn (Y.G.); 2 Institute of Clinical Pharmacology, Hunan Key Laboratory of Pharmacogenetics, Central South University, Changsha 410078, China; 3 Department of Oncology, Changsha Central Hospital, Changsha 410006, China; chenshuhui2008@126.com; 4 Department of Pharmacy, Xiangya Hospital, Central South University, Changsha 410008, China; quqiang1983@Sina.com; 5 State Key Laboratory of Ophthalmology, Zhongshan Ophthalmic Center, Sun Yat-sen University, Guangzhou 510275, China; kjade.cheng@hotmail.com

**Keywords:** long non-coding RNA, AB073614, glioma, prognostic biomarker

## Abstract

Dysregulated long noncoding RNAs (lncRNAs) have been found in human diseases, especially in cancer. Emerging evidence indicates that dysregulated lncRNAs are implicated in tumorigenesis and cancer progression. LncRNA AB073614 characterized as a new candidate lncRNA promotes the development of ovarian cancer. However, the role of lncRNA AB073614 in human gliomas remains unknown. The expression of AB073614 was detected in 65 glioma tissues and 13 normal brain tissues by qRT-PCR, showing that lncRNA AB073614 expression was significantly up-regulated in cancerous tissues compared with normal brain tissues (*p* < 0.001), and it was positively correlated with tumor grade (I–II grades *vs.* III–IV grades, *p* = 0.013) in glioma patients. Kaplan-Meier analysis demonstrated that increased AB073614 expression contributed to poor overall survival (HR (hazard ratio) = 1.952, 95%CI: 1.202–3.940, *p* = 0.0129). Further, univariate Cox regression analysis indicated that lncRNA AB073614 overexpression was an unfavorable prognostic factor in gliomas (HR = 1.997, 95%CI: 1.135–3.514, *p* = 0.016), regardless of the tumor grade (I–II grades *vs.* III–IV grades, HR = 1.902, 95%CI: 1.066–3.391, *p* = 0.029). Finally, after adjustment with age, sex, tumor grade and tumor location, multivariate Cox regression analysis suggested that both highly expressed lncRNA AB073614 (HR = 2.606, 95%CI: 1.408–4.824, *p* = 0.002) and high tumor grade (III–IV grades, HR = 2.720, 95%CI: 1.401–5.282, *p* = 0.003) could be considered independent poor prognostic indicators for glioma patients. In conclusion, our study suggested that increased lncRNA AB073614 expression may be identified as a poor prognostic biomarker in gliomas.

## 1. Introduction

Gliomas account for the great majority of primary tumors in the brain [1]. Within each histological subtype, they could be further categorized into four grades: I–IV lesions based on the degree of malignancy [2]. Unfortunately, patients diagnosed with glioblastoma (GBM) in the brain have a poor prognosis with the median survival time of only 12 to 15 months. Despite the development of multimodal and aggressive treatments including surgical resection, chemotherapy, and radiation therapy in the past decades, the outcomes of GBM patients remain unsatisfactory [3]. Therefore, it is imperative to comprehend the pathogenic mechanism of GBM and identify new biomarkers and therapeutic targets for GBM patients [4,5]. 

Long noncoding RNAs (lncRNAs) are commonly considered as nonprotein coding transcripts longer than 200 nucleotides. With the development of biocomputational research tools such as lncRNAdb, ChIPBase, LNCipeida and lncRNAtor, the number of lncRNAs being identified is rapidly increasing [6,7]. Though thousands of human lncRNAs have been identified, only a small proportion of them have been functionally characterized in detail. Previous researches have shown that lncRNAs widely participate in the regulation of critical cellular functions, including transcriptional, posttranscriptional, and epigenetic mechanisms of gene regulation [8,9].

Recently, the relationship between lncRNAs and tumors have gained wide attention. Accumulating evidence indicates that lncRNAs may play a critical role in the development and progression of various cancers, including breast cancer [10], gastric cancer [11,12,13,14], bladder cancer [15], lung cancer [16], and colorectal cancer [9]. Recent evidence indicates that some lncRNAs, with their significance in brain evolution, development and diseases, could be used as potential biomarkers and therapeutic targets for gliomas [17,18,19]. It is noteworthy that some lncRNAs have been found to be differentially expressed in glioma samples and normal brain tissues [6,7,20]. It has been reported that lncRNAs may take part in regulating certain tumorigenic processes in glioma, such as cellular proliferation and cell cycle progression [21,22]. However, the precise mechanism underlying how lncRNAs promote or suppress tumorigenesis remains largely unknown. Zhang *et al.* [21] found that lncRNA HOTAIR (Hox transcript antisense intergenic RNA) promoted cell cycle progression of GBM in an EZH2 (enhancer of zeste homolog 2) dependent manner. Also, the study showed that HOTAIR might be related to gene methylation via the HOTAIR 5’ domain-EZH2 axis. Another research showed that miR-21 could directly bind to lncRNA CASC2 (cancer susceptibility candidate 2) by the putative miRNA response element [20]. It was found that lncRNA H19 played a trigger role in glioma cell invasion by directly regulating miR-675 expression [23]. On the other hand, some lncRNAs, such as lncRNA CASC2, have been characterized as tumor suppressors in glioma [24]. However, the exact potential mechanisms remain not fully elucidated [23]. Cheng *et al.* found that lncRNA AB073614 promoted tumorigenesis and predicted a poor prognosis in ovarian cancer [25], while its role in glioma is still unclear. In this study, we found that lncRNA AB073614 expression was significantly higher in glioma tissues compared with normal brain tissues, and the increased AB073614 expression was closely associated with poor outcomes of glioma patients.

## 2. Materials and Methods

### 2.1. Clinical Samples and Data Collection

The glioma samples were acquired from 65 GBM patients who underwent radical resection between November 2010 and June 2013 at the First Affiliated Hospital of Nanchang University (Nanchang, Jiangxi, China). 13 healthy control brain specimens were obtained from trauma/epilepsy surgery. The brain samples were immediately frozen in liquid nitrogen and stored at −80 °C until RNA extraction. None of these samples were collected after any anticancer treatments including chemotherapy, radiotherapy and surgery and no bias against the selection for the glioma samples was introduced in this study. Overall survival (OS) was defined as the interval between the dates of surgery and death and all patients had experienced a follow-up period lasting 48 months since the date of surgical resection. This study was approved by the Research Ethics Committee of First Affiliated Hospital of Nanchang University (Ethical Approval No. 2010-015; Date: 12 March 2010) and written informed consent was obtained from each patient.

### 2.2. Total RNA Extraction and Quantitative RT-PCR

Total RNA was extracted from tissues using Trizol reagent (Invitrogen, San Diego, CA, USA) according to the manufacturer’s instructions before being dissolved in 20 μL diethylpyrocarbonate-treated water. Then, quantitative real-time polymerase chain reaction (qRT-PCR) was carried out using a LightCycler480 System (Roche Diagnostics Ltd., Rotkreuz, Switzerland) and SYBR Green real-time PCR Kit produced by Takara Biotechnology (Takara, Dalian, China) to detect the expression of lncRNA AB073614, with GAPDH as a normalizing control. The primer sequences were as follows. The primers for AB073614 were 5’-TCTGCTCCTGGGTCTTACAC-3’ and 5’-TGCAACCACATGTAACCACA-3’; the primers for GAPDH were 5’-CCCATCACCATCTTCCAGGAG-3’ and 5’-GTTGTCATGGATGACCTTGGC-3’. The qRT-PCR amplification was performed in triplicate starting at 95 °C for 10 min, followed by 40 cycles at 95 °C for 10 s, and 60 °C for 60 s. The relative expression of lncRNA AB073614 was calculated and normalized using the delta-delta CT (2^−ΔΔCt^) method relative to GAPDH.

### 2.3. Statistical Analysis

SPSS 20.0 software system (IBM, SPSS, Chicago, IL, USA) was used for statistical analysis. All data were represented as means ± standard deviation (S.D.). A Student’s *t* test was applied to analyze the differences in AB073614 levels between GBM tissues and normal brain tissues. Pearson’s Chi-square test or Fisher’s exact test was performed to determine the relationship between lncRNA AB073614 expression levels and clinicopathological characteristics. Kaplan-Meier method was used to evaluate the overall survival rates, and the log-rank test was performed to calculate the difference in survival. The Cox proportional hazards regression model was used for univariate and multivariate analyses to assess the effects of the clinicopathological variables and AB073614 mRNA expression levels on overall survival. A *p*-value of <0.05 was considered statistically significant.

## 3. Results

### 3.1. AB073614 Is Up-Regulated in Glioma Tissues

**Figure 1:**
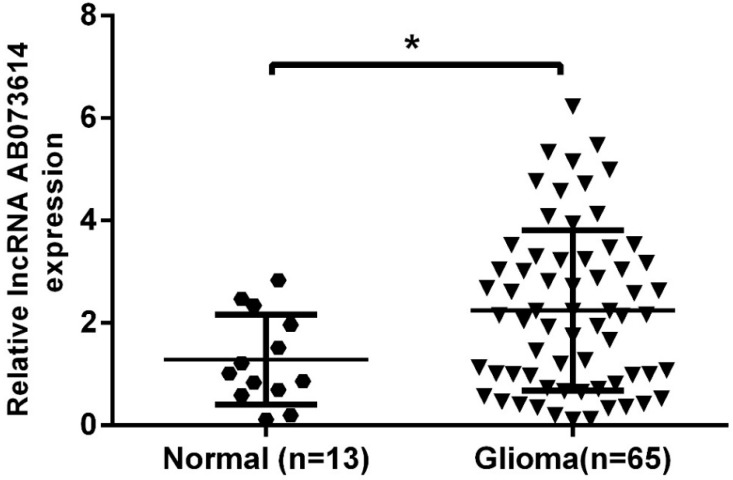
AB073614 was overexpressed in glioma tissues. The expression levels of AB073614 in 65 glioma and 13 normal brain tissues were detected by quantitative real-time polymerase chain reaction (qRT-PCR). AB073614 expression level was normalized to that of GAPDH (* *p* < 0.001).

It was reported that AB073614 was greatly up-regulated in human ovarian cancer compared with the normal counterparts [25]. To determine the role of this lncRNA played in glioma, we detected the expression of AB073614 in glioma tissues by qRT-PCR, finding that AB073614 level was significantly increased in glioma tissues compared with the normal brain tissues (*p* < 0.001) (Figure 1). 

### 3.2. Relationship between lncRNA AB073614 Expression and Clinicopathological Characteristics in Glioma Patients

To further explore the role of AB073614 in determining the clinical significance of glioma, we analyzed the association between AB073614 expression and clinicopathological features in 65 glioma patients, discovering that AB073614 was more highly expressed in high-grade than low-grade glioma tissues (*p* = 0.013) (Table 1). However, AB073614 expression was not associated with gender, age or tumor location (*p* = 0.591, 0.407 and 0.275, respectively) (Table 1). Taken together, these data suggest that AB073614 could play an important role in the progression of glioma.

### 3.3. High Level of lncRNA AB073614 mRNA Were Significantly Associated with Decreased Overall Survival of Glioma Patients

**Figure 2:**
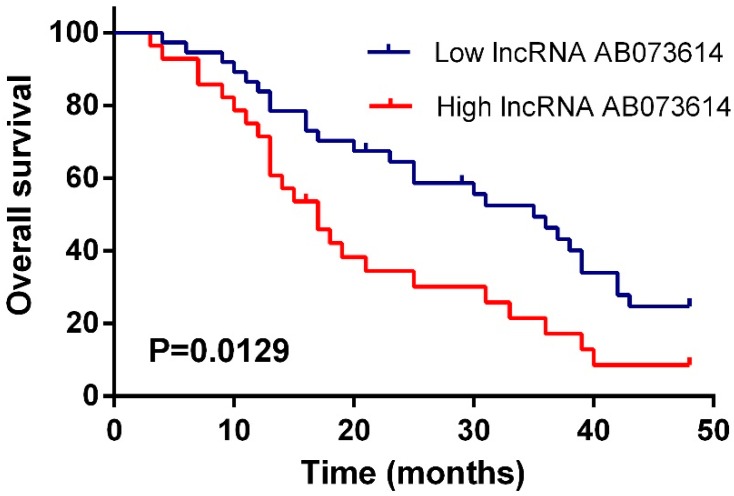
Overexpression of AB073614 indicated an unfavorable prognosis. Kaplan-Meier survival analysis of overall survival in 65 patients based on AB073614 expression status.

In order to assess the prognostic value of lncRNA AB073614 expression for glioma, we investigated the association between lncRNA AB073614 expression levels and OS through Kaplan-Meier analysis with log-rank test in 65 glioma cases, observing that AB073614 expression was significantly associated with the overall survival of glioma (HR (hazard ratio) = 1.952, 95%CI: 1.202–3.940, *p* = 0.0129) (Figure 2).

### 3.4. Level of lncRNA AB073614 mRNA Is a Potential Prognostic Marker for Glioma Patients

Univariate analysis using a Cox proportional hazards model to evaluate the potential of using AB073614 mRNA expression level as a prognostic marker for glioma patients showed that both AB073614 overexpression (HR = 1.997, 95%CI: 1.135–3.514, *p* = 0.016) and the tumor grade (HR = 1.902, 95%CI: 1.066–3.391, *p* = 0.029) were the prime variables for glioma prognosis (Table 2). After adjusting for clinicopathologic variables, AB073614 overexpression ( HR = 2.606, 95%CI: 1.408–4.824, *p* = 0.002) and the tumor grade ( HR = 2.720, 95%CI: 1.401–5.282, *p* = 0.003) remained significantly correlated with the prognosis of glioma patients (Table 2). 

## 4. Discussion

LncRNAs are commonly defined as RNA molecules transcribed from various genomic locations, including the promoters, enhancers, introns, or antisense coding regions of genes, or their own stand-alone position in the genome. Accumulating evidence suggests that lncRNAs play an important role in cellular development and human diseases, especially in cancers [26]. Recently, an increasing number of studies have reported a close association between lncRNA expression and tumorinegesis and prognosis [27,28,29]. LncRNAs may regulate certain tumorigenic processes in glioma such as cellular proliferation, cell cycle progression and invasion, advocating the usage of lncRNAs as novel biomarkers and therapeutic targets for gliomas [21,22]. In addition, some lncRNAs, such as lncRNA CASC2, TUG1, TSLC1-AS1, have been characterized as tumor suppressors in glioma [23,30,31].

At present, several long noncoding RNAs have been reported to be directly involved in glioma initiation and development [32,33,34], and mechanistic studies revealed that lncRNAs expression could be modulated by various factors in glioma cells, including the mTOR signaling pathway [35], transcription factors [21] and mRNA interactions [34]. Zhang *et al.* [21] discovered that lncRNA HOTAIR promoted glioma development and indicated a poor prognosis by regulating cell cycle progression through EZH2. Wang *et al.* [36] found that lncRNA HOXA11-AS played an important role in glioma grade and poor prognosis and act as an independent prognostic factor in glioblastoma multiforme patients via the regulation of proliferation and cell cycle of GBM cells by affecting the expression of P16, P21 and P27. However, so far, only a handful of lncRNAs have been found to be involved in glioma oncogenesis. New promising approaches for glioma treatment may emerge with the identification of novel glioma-associated lncRNAs and investigation of their clinical significance and functions [37]. LncRNA AB073614 is a novel lncRNA transcript that first be identified as a Homo sapiens primary hepatoblastoma cDNA [38]. Cheng *et al.* found that lncRNA AB073614 promoted tumorigenesis and predicted poor prognosis in ovarian cancer by targeting the AKT-ERK1/2 signaling pathway [25]. So far, no study has reported the role of lncRNA AB073614 in any other cancer, such as glioma. 

In this study, for the first time, we investigated the association of lncRNA AB073614 expression with glioma progression and prognosis. We found that the levels of lncRNA AB073614 in glioma patients were significantly higher than those in normal subjects. However, no association of AB073614 expression with gender, age or tumor location was observed. Importantly, high AB073614 expression was correlated with lower overall survival rate and could serve as an independent prognostic factor in patients with glioma. Taken together, these findings indicate that lncRNA AB073614 also acts as a functional cancer gene in glioma development, which is consistent with the research in ovarian cancer conducted by Cheng *et al.* [25]. However, the precise role of lncRNA AB073614 in development and progression of glioma remains to be elucidated, and further investigations in cell and animal models will be needed.

## 5. Conclusions

In conclusion, the present study has shown for the first time that lncRNA AB073614 is overexpressed in glioma tissues and that its up-regulation may be indicative of poor survival rates and a higher risk for brain cancer progression, and it could have a potential role as a prognostic marker in glioma patients. 

## Figures and Tables

**Table 1:** Correlation of lncRNA AB073614 expression with clinicopathological features in glioma patients.

Clinical Characteristic	Number of Patients	Number of Patients		*p*-Value
		High Expression	Low Expression	
Age (year)				
<45	36	17	19	0.591
≥45	29	11	18	
Sex				
Male	38	18	20	0.407
Female	27	10	17	
Tumor Grade				
Low grades I–II	30	8	22	**0.013** *
High grades III–IV	35	20	15	
Tumor location				
Frontal	19	6	13	0.275
Parietal	5	2	3	
Occipital	13	5	8	
Temporal	17	7	10	
Others	11	8	3	

* The values had statistically significant differences.

**Table 2:** Univariate and multivariate analyses of different clinicopathological variables and lncRNA AB073614 expression.

Variable	Univariate Analysis		Multivariate Analysis	
	HR (95% CI)	*p*-Value	HR (95% CI)	*p*-Value
Age (<45 *vs.* ≥45 years)	1.352 (0.768–2.383)	0.296	1.201 (0.641–2.252)	0.567
Sex (male *vs.* female)	1.190 (0.675–2.097)	0.547	0.859 (0.458–1.610)	0.635
Tumor Grade (I–II *vs.* III–IV)	1.902 (1.066–3.391)	**0.029** *	2.720 (1.401–5.282)	**0.003** *
Tumor Location		0.754		0.413
Parietal *vs.* Frontal	0.678 (0.301–1.528)	0.349	0.439 (0.121–1.588)	0.209
Occipital *vs.* Frontal	0.458 (0.126–1.670)	0.237	0.876 (0.366–2.095)	0.766
Temporal *vs.* Frontal	0.711 (0.295–1.711)	0.447	0.886 (0.410–1.916)	0.759
Others *vs.* Frontal	0.641 (0.281–1.466)	0.292	1.542 (0.667–3.565)	0.311
AB073614 Expression (low *vs.* high)	1.997 (1.135–3.514)	**0.016** *	2.606 (1.408–4.824)	**0.002** *

* The values had statistically significant differences.
